# Comparison of endometrial regenerative cells and bone marrow stromal cells

**DOI:** 10.1186/1479-5876-10-207

**Published:** 2012-10-05

**Authors:** Huan Wang, Ping Jin, Marianna Sabatino, Jiaqiang Ren, Sara Civini, Vladimir Bogin, Thomas E Ichim, David F Stroncek

**Affiliations:** 1 Department of Transfusion Medicine, Clinical Center, National Institutes of Health, Bethesda, MD, USA; 2 MediStem Inc, San Diego, CA, USA; 3 Department of Transfusion Medicine, Cell Processing Section, 10 Center Drive-MSC-1288, Building 10, Room 3C720, Bethesda, MD, 20892-1288, USA

**Keywords:** Endometrial regenerative cells, Bone marrow stromal cells, Mesenchymal stromal cells, Mesenchymal stem cells, Regenerative medicine

## Abstract

**Background:**

Endometrial regenerative cells (ERC) and bone marrow stromal cells (BMSC) are being used in clinical trials. While they have been reported to have similar characteristics, they have not been directly compared.

**Methods:**

We compared micro RNA (miRNA) and gene expression profiles, soluble cytokine and growth factor levels and ability to inhibit ongoing mixed leukocyte reaction (MLR) of ERC and BMSC each derived from 6 healthy subjects.

**Results:**

ERC and BMSC miRNA and gene expression profiles were similar, but not identical; more differences were noted in the expression of genes than in miRNAs. Genes overexpressed in ERCs were more likely to be in immune and inflammation pathways and those overexpressed in BMSCs were more likely to be in stem cell and cancer signaling pathways. In addition, the levels of IL-8 and ICAM-1 were greater in ERC supernatants while the levels of HGF, VEGF, IL-6, CXCL12, TGFB1 and TGFB2 were greater in BMSC supernatants. Additionally, ERC demonstrated greater inhibition of the proliferation of mixed leukocyte cultures.

**Conclusions:**

These results suggest that the in vivo effects of ERC and BMSC may differ. Multiple properties of stromal cells are responsible for their in vivo effectiveness and ERC may be more effective for some of the clinical applications and BMSC for others. Studies in animal models or clinical trials will be required to more fully characterize the differences between ERC and BMSC.

## Background

Mesenchymal stem cells (MSC) have been the subject of numerous studies for their ability to differentiate into various specialized cells and their great therapeutic potential, particularly in tissue regeneration. These cells have been isolated from many different tissues. One of the most commonly investigated MSC are those derived from bone marrow, which are known as bone marrow stromal cells (BMSC). These are fibroblast-like, plastic adherent cells from the bone marrow [1] that express CD73, CD90 and CD105 [2,3]. These multipotent cells have the ability to differentiate into osteoblast, chondrocyte, and adipocyte colonies [4,5] and have been shown to be capable of bone regeneration [6,7], effective in treating acute graft-versus-host-disease (GVHD) [8-10] and have been investigated to treat other diseases such as cirrhosis [11], ischemic heart disease [12], Crohn’s Disease [13] and autoimmune disorders.

Meng and collaborators [14] have isolated a type of stromal cell from menstrual blood, Endometrial Regenerative Cells (ERC), which also have promising clinical potential. These cells are not specifically classified as MSC, though they do express most MSC markers such as CD9, CD29, CD41a, CD44, CD59, CD73, CD90, and CD105. ERC are distinct from previously characterized endometrial stromal cells, or endometrial mesenchymal stem cells, in that they do not express the BMSC marker STRO-1 [14,15]. They can differentiate into tissue types beyond bone, fat, and cartilage and therefore may be pluripotent to some degree [14,15]. Studies in animal models also suggest that ERC can be used to treat critical limb ischemia via stimulation of angiogenesis [15], inhibit glioma growth and reduce tumor neovascularization and possess immunosuppressive properties [15,16]. Furthermore, unlike embryonic stem cells, ERC do not appear to pose the risk of teratoma development in vivo [14], and they are easily collected and expanded. A phase I clinical study performed on four multiple sclerosis patients showed that ERC could be used allogenically and safely, and the treatment seemed to have prevented disease progression [17]. In addition, the FDA has granted the approval for ERC to be used in additional human clinical trials. Although early evidence shows a promising future for the allogeneic use of ERC in human patients, it is nevertheless necessary to confirm and expand our current understanding.

We compared ERC and BMSC by examining their morphology, cytokine production, inhibition of mixed leukocyte reactions (MLRs), micro RNA (miRNA) expression and global gene expression. We hypothesized that the two types of cells would exhibit different properties which could result in distinct functional and clinical properties.

## Materials and methods

### Study design

Six different ERC samples and six different BMSC samples from individual donors were chosen and culture for 6–7 days. Since the media normally used to support the growth of ERC and BMSC were different, we grew the ERC in both media to determine if any differences between ERC and BMSC were due to culture medium type. We compared ERC and BMSC by analyzing the global gene expression and miRNA profiling, cytokine production, morphology and inhibition of MLR. Hematopoietic stem cell (HSC), embryonic stem cell (ESC) and fibroblast lines were utilized as controls in miRNA and global gene expression comparisons. This study was approved by the NHLBI Institutional Review Board.

### Cell lines

The ERC were provided by MediStem (MediStem Inc., San Diego, CA, USA), BMSC were obtained from marrow aspirates of healthy subjects (Cell Processing Section, Department of Transfusion Medicine, Clinical Center, NIH Bethesda, MD, USA), the fibroblasts were CRL2429, CRL2352 (ATCC, Manassas, VA, USA) and NuFF1(Global Stem Inc, Rockville, MD, USA), the ESC were H9 (WiCell Research Institute, Madison, WI, USA), I6 (Technion-Israel Institute of Technology, Haifa, Israel) and BG01v (ATCC), the HSC were CD34^+^ cells were isolated with monoclonal antibodies and paramagnetic beads from G-CSF-mobilized peripheral blood stem cell concentrates (Cell Processing Section, NIH).

### Culture of ERC

Six frozen human ERC lines were thawed from liquid nitrogen, washed, and manually counted. Passage 1 cells were first expanded to Passage 2 in Modified Eagle Medium with Nutrient Mixture F-12 with 10% fetal bovine serum and 10 μg gentamicin (ERC medium). Passage 2 cells were then plated on two 75 cm^2^ culture flasks at a density of approximately 3x10^3^cells/cm^2^ in two types of media: ERC Medium and alpha Minimum Essential Medium with 20% fetal bovine serum and 10 μg gentamicin (BMSC medium). Cells were observed for adherence after 24 h and culture medium was changed after 3 days. Once cells reached >80% confluence (6–7 days), they were harvested for analysis. Two to three million cells were lysed in 1 ml Trizol and 3 ml of supernatant was collected for proteomic analysis. Cells cultured in ERC media were designated ERC-E and in BMSC media ERC-B.

### Culture of BMSC

Frozen human BMSC isolated from bone marrow aspirates were thawed from liquid nitrogen, washed, and manually counted. Passage 2 BMSC were plated on 75 cm^2^ flasks at a density of 3x10^3^cells/cm^2^ in BMSC medium and observed for adherence after 24 h. Culture medium was changed after 3 days. Passage 3 cells were harvested at >80% confluence after 6–7 days for further analysis. Two to three million cells were lysed in 1 ml Trizol and 3 ml of supernatant were collected for proteomic analysis.

### Cell imaging of embryonic stem cell marker analysis

BMSC and ERC at passage 2 were thawed and cultured in BMSC medium at a density of 1x10^5^ cells per well in flat bottom six-well plates. Cell medium was changed after 3 days and light microscopy images were taken for both cell types. Cell medium was aspirated after 6 days of culture; cells were washed with 1X PBS and fixed with 4% paraformaldehyde. Cells were permeabilized with 1% saponin in PBS and were stained for the following embryonic stem cell markers: Oct-4, Nanog, SSEA-4, SSEA-1, Klf-4, and TRA-1-81 at room temperature overnight. Cells were rinsed with 1% saponin and incubated with anti-tubulin for 1 hour, washed with 1% saponin, and rinsed with 1X PBS before observation by immunofluorescence microscopy (Zeiss Axio Observer Inverted Microscope Carl Ziess, Gottingen, Germany).

### Measuring cytokine and growth factor levels with proteome profiler antibody arrays

Supernatants from 3 ERC samples (ERC-B) and 3 BMSC samples cultured in BMSC medium and 3 BMSC samples cultured in the same medium were analyzed for soluble proteins and cytokines related to immunomodulation using the Proteome Profiler Human Cytokine Array Kit-Panel A (R&D Systems, Minneapolis, MN, USA), which detects most common immunomodulatory cytokines. Each supernatant sample was incubated with a cytokine panel membrane according to manufacturer’s protocol with modifications. Incubation times were doubled to optimize signal detection. The membranes were scanned (ChemiDocsTM XRS + plus ImageLab) and analyzed with Bio-Rad Image Lab software (Bio-Rad Laboratories, Hercules, CA, USA). Semi-quantitative analysis was performed by measuring the average pixel intensity of each array, normalizing the values to the intensity of the positive control, and subtracting the background. The sample intensity to control ratio was the average of the 3 ERC samples and 3 BMSC samples, respectively.

### Supernatant cytokine and growth factor analysis using searchlight protein array

Three ERC-B and 3 BMSC supernatant samples were evaluated for cytokine concentrations using SearchLight Protein Array Analysis (Aushon Biosystems, Billerica, MA, USA). The cytokines and growth factors measured were: human fibroblast growth factor beta (FGFb), hepatocyte growth factor (HGF), platelet-derived growth factor-BB (PDGFBB), Vascular endothelial growth factor (VEGF), Interleukin 10 (IL-10), Interleukin 6 (IL-6), chemokine (C-X-C) ligand 12 (CXCL12), Transforming growth factor beta 1 (TGFB1), and Transforming growth factor beta 2 (TGFB2).

### Mixed leukocyte reaction inhibition

The immunosuppressive properties of BMSC and ERC were compared using an MLR assay (SAIC-Frederic, Frederic, MD). Ficoll-separated peripheral blood mononuclear cells (PBMC) were plated in 96-well plates at 1x10^5^ responders per well. Responders were co-cultured with 2500 cGy irradiated stimulator PBMC at a concentration of 1x10^5^ cells/well. BMSC and ERC were added at the following concentrations, 10^4^ and 10^5^ cells/well. Culture plates were incubated for 6 days in a humidified 5% CO_2_ incubator at 37°C. On the day of harvest, 0.5 μCi of 3 H-thymidine (3 H-TdR) was added to each well for 4 hours with lymphocyte proliferation measured using a liquid scintillation counter. The effect of BMSC on MLR was calculated as the percentage of the suppression compared with the proliferative response of the positive control without BMSC, where the positive control was set to 0% suppression. The experiments were performed three times for each variable described.

### Gene expression analysis

Total RNA from ERC, BMSC, fibroblast, ESC, and HSC samples were extracted using miRNA Easy Kit (Qiagen, Valencia, CA, USA). RNA concentration was measured using ND-8000 spectrophotometer (Nano Drop Technologies, Wilmington, DE, USA).

Microarray expression experiments were performed on 4 × 44 K Whole Human Genome Microarray (Agilent technologies, Santa Clara, CA, USA), according to the manufacturer's instructions. The Universal Human Reference RNA (Stratagene, Santa Clara, CA, USA) was co-hybridized with each sample. Images of the arrays were acquired using a microarray scanner G2505B (Agilent technologies) and image analysis was performed using feature extraction software version 9.5 (Agilent Technologies). The Agilent GE2-v5_95 protocol was applied using default settings. The resulting data files were either uploaded to the mAdb database ( http://nciarray.nci.nih.gov/) and further analyzed using BRBArrayTools [18] developed by the Biometric Research Branch, National Cancer Institute ( http://linus.nci.nih.gov/BRB-ArrayTools.html) or imported and analyzed using Partek Genomics Suite 6.5 (Partek, Inc, St Louis, MO). Hierarchical cluster analysis and TreeView software were used to visualize the data, and Ingenuity Pathway Analysis (IPA, Ingenuity Systems, Redwood City, CA, USA) was used to perform gene pathway analysis.

### Quantitative reverse transcription real time PCR (qRT-PCR)

Quantitative RT-PCR was performed to confirm the expression of selected genes differentially expressed between ERC and BMSC as determined by microarray studies. One μg of total RNA from 6 BMSC, 6 ERC-B, 6 ERC-E, 3 Fibroblast, 2 ESC, and 2 HSC samples were reverse transcripted (RT) with random hexamer primer and the RT product was further diluted for TaqMan Gene Expression Assays (Applied Biosystems, Carlsbad, CA, USA) using standard settings. The genes selected were tumor necrosis factor superfamily 4 (TNFSF-4,), interleukin 8 (IL-8), intercellular adhesion molecule 1 (ICAM-1), vascular cell adhesion molecule 1 (VCAM-1), integrin alpha 10 (ITGA-10), prostaglandin-endoperoxide synthase 2 (PTGS2), and matrix metallopeptidase 3(MMP3). Gene expressions were quantified with TaqMan Gene Expression Assay of each above listed gene (Assay IDs: Hs00182411; Hs99999034_m1; Hs00164932_m1; Hs01003372_m1; Hs00174623_m1; Hs00153133_m1; Hs00968305 respectively, Applied BioSystems, Life Technologies, Santa Clara, CA, USA). All reactions were run in duplicate. Real-time PCR was performed using ABI 7900HT Real-time PCR system. The data was acquired using ABI SDS v2.3 software determining the threshold cycle (Ct) by normalizing to the endogenous control 18 s RNA (Hs99999901_s1, Applied Biosystems). The fold change of each gene against the calibrator was calculated using the equation 2^-ΔΔCt^.

### miRNA expression analysis

MiRNA profiling was performed on Agilent’s 8x15K human miRNA microarray chips. Total RNA from 4 ERC-B, 3 BMSC, 3 fibroblast, 3 ESC, and 3 HSC samples were labeled and hybridized according to manufacturer’s instruction. Slides were scanned using Agilent Microarray Scanner Version C, and data were extracted using Feature Extraction software version 11.0 (Agilent Technology). Data analysis was performed by using Partek Genomics Suite 6.5 (Partek, Inc, MO).

## Results

### Cell morphology, growth and stem cell marker expression

**Figure 1:**
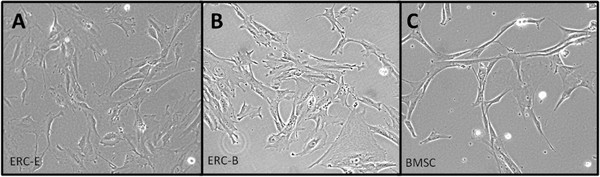
**Comparison of ERC and BMSC morphology in various culture conditions.A**: Early passage (P3) ERC cultured in ERC medium (10 % FBS in DMEM F-12). **B**: Early passage (P3) ERC from the same donor cultured in BMSC medium (20 % FBS in αMEM). **C**: Early passage (P3) BMSC cultured in BMSC medium.

**Figure 2:**
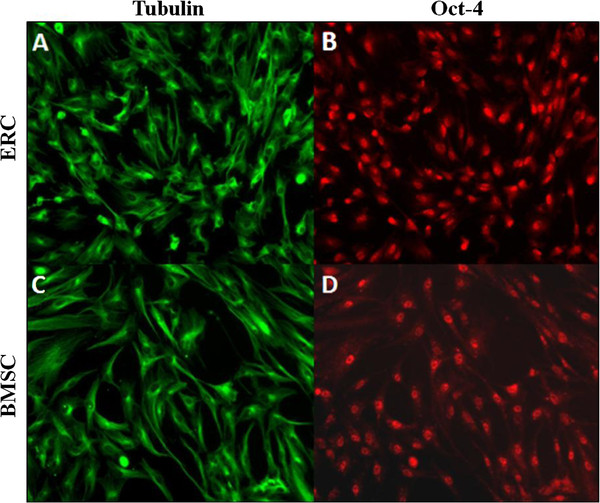
**Immunofluorescence staining of ERC and BMSC.A**-**B**: ERC were labeled for tubulin (**A**) and the embryonic stem cell marker Oct-4 (**B**), 10X magnification. **C**-**D**: Immunofluorescence staining of BMSC for tubulin (**C**) and Oct-4 (**D**), 10X magnification.

ERC were cultured in both BMSC medium (ERC-B) and a modified ERC medium based on the formulation used by Medistem, Inc (ERC-E). We hypothesized that the growth rate and morphology of ERC would not be affected by the difference in tissue culture medium. Population doubling time (PDT) was calculated revealing that ERC-B had a slightly shorter average PDT when compared with ERC-E (Table 1); however, no morphological differences were observed for ERC cultured in different media (Figure 1A,B). When compared with BMSC, ERC had a longer PDT (Table 1), larger cytoplasmic area and were less spindly (Figure 1 A-C). Immunofluorescence imaging revealed that both ERC (Figure 2A,B) and BMSC (Figure 2C,D) were positive for Oct-4 while both types of cells were found to be negative for SSEA1, SSEA4, Nanog, TRA-1-81, and Klf-4 (data not shown).

**Table 1:** Population doubling time of ERC and BMSC

**Cell Type**	**Culture Media**	**Number**	**PDT (hours)**
ERC	BMSC Medium	7	42.8
ERC	ERC Medium	7	47.6
BMSC	BMSC Medium	4	40.3

PDT = population doubling time.

### Soluble protein production

Soluble cytokines and growth factors expressed by ERC were examined and compared to those expressed by BMSC. ERC supernatant was taken from ERC-B to minimize the differences due to culture medium. ERC and BMSC produced many of the same cytokines albeit at varying levels (Table 2). Among the proteins with high signal intensities IL-8 and soluble ICAM-1 (CD54) were notably higher in ERC supernatant than that of BMSC (Table 2). We also measured the concentration of proteins which were reported to provide some of the beneficial effects of BMSCs [19] using a multiplex ELISA platform (Searchlight) and found that BMSC supernatants had higher concentrations of HGF, VEGF, IL-6, CXCL12, TGFB1 and TGFB2 than ERC supernatants (Table 3). bFGF, PDGFBB and IL-10 levels were too low to be detected in either group.

**Table 2:** Mean pixel intensity values from cytokine array panels of BMSC and ERC-B supernatants

Cell Type^*^	**CXCL1**	**sICAM-1**	**IL-8**	**CCL2**	**MIF**	**Serpin E1**
BMSC 1	64.1	0.5	105.8	154.0	427.4	359.6
BMSC 2	35.2	0.0	89.4	1.3	175.2	222.4
BMSC 3	20.7	0.0	23.2	1.3	174.5	169.1
Mean ± 1SD	40.0 ± 22.1	0.2 ± 0.3	72.8 ± 43.7	52.2 ± 88.2	259.0 ± 145.8	250.4 ± 98.3
ERC 1	43.2	40.8	903.7	16.9	434.7	333.5
ERC 2	2.0	1.7	152.3	0.0	24.0	239.8
ERC 3	24.3	46.7	236.2	0.4	109.1	161.9
Mean ± 1SD	23.2 ± 20.6	29.7 ± 24.5	430.7 ± 411.8	5.8 ± 9.6	189.3 ± 216.8	245.1 ± 85.9

*Three BMSC and 3 ERC different donors were analyzed.

**Table 3:** Detected levels of cytokines in BMSC and ERC-B supernatants (pg/ml)

Cell Type^*^	**HGF**	**VEGF**	**IL-6**	**CXCL12**	**TGFB1**	**TGFB2**
BMSC1	3344	31425	84151	9605	33659	12466
BMSC2	538	13345	10596	21072	38135	6766
BMSC3	617	10013	20350	8161	31963	3873
Mean ± 1SD	1500 ± 1598	18261 ± 11521	38366 ± 39950	12946 ± 7074	34586 ± 3189	7701 ± 4372
ERC1	224	4	449	7093	20282	2933
ERC2	44	13	108	540	19294	2934
ERC3	216	0.0	1394	12249	25577	2747
Mean ± 1SD	161 ± 102	5.7 ± 6.6	650 ± 666	6627 ± 5868	21718 ± 3379	2871 ± 108
BMSC CM	15.2	6.5	0.1	8.5	12014.6	1546.1

*Three BMSC and 3 ERC donors were analyzed.

*BMSC CM* = Bone marrow stromal cell culture media.

### Mixed leukocyte reaction inhibition

**Figure 3:**
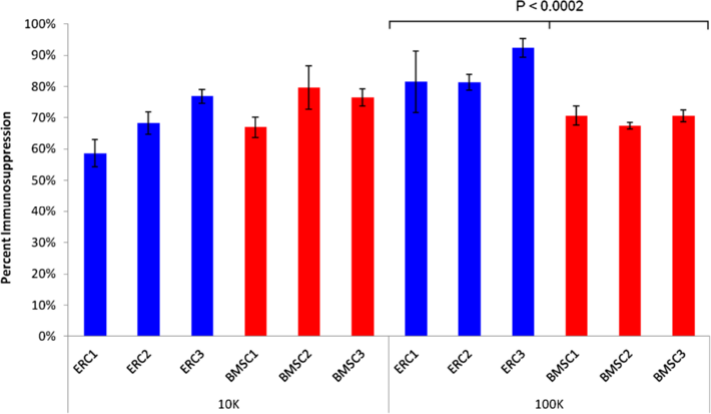
**Immunosuppression of T cell responses in mixed lymphocyte reactions (MLRs) by ERC-B and BMSC.** The bars indicate responder T cell proliferation when incubated with irradiated T cell stimulator cells and ERC-B or BMSC. Two doses of ERC-B and BMSC were tested: 10,000 and 100,000 cells. The measures were performed in triplicate and converted to percent immunosuppression by normalizing to the proliferation of T cells without BMSC co-incubation.

The immunosuppressive capabilities of both ERC and BMSC were investigated by MLR inhibition. When responder T cells were stimulated in the presence of ERC-B and BMSC at a concentration of 10,000 cells per well, the BMSC were generally more immunosuppressive. However, a two-sample equal variable *T*-test revealed that this difference was not statistically significant (Figure 3). When responder T cells were stimulated in the presence of ERC-B and BMSC at a concentration of 100,000 cells per well, ERC-B were significantly more immunosuppressive (*P* < 0.0002).

### Gene expression, class comparison and pathway analysis

**Figure 4:**
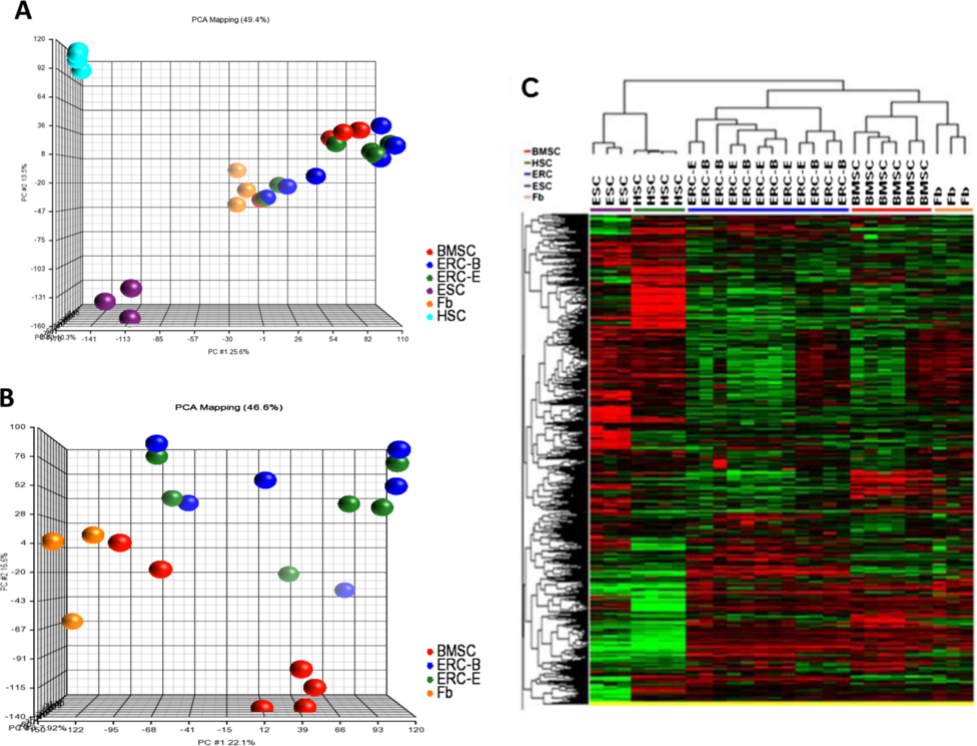
**Principal Component Analysis (PCA) and hierarchical clustering analysis of differentially expressed genes among ERC-E, ERC-B, BMSC, HSC, ERC, and fibroblast (Fb) samples.A**: Principal component analysis of all six cell types based on differentially expressed genes. **B**: PCA analysis comparing ERC-E, ERC-B, BMSC and fibroblast (Fb) samples using the differentially expressed genes. **C**: Unsupervised clustering of all samples based on the differentially expressed genes.

Global gene expression analysis was used to profile the ERC-E, ERC-B, BMSC, HSC, fibroblasts, and ESC. Principal Component Analysis (PCA) performed on the entire dataset of 34,127 genes revealed that the samples formed four distinct clusters (Figure 4A). The HSC were in one cluster and ESC in a second; both clusters were located far from each other and from the rest of the samples. Another cluster was made up of fibroblasts which was closer to the fourth cluster made up of the ERC-E, ERC-B and BMSC. This suggested that ERC and BMSC were similar to each other and they were more similar to fibroblasts than to ESC or HSC from a global perspective. There were, however, some differences between ERC and BMSC. A PCA analysis of fibroblast, ERC-E, ERC-B, and BMSC samples with HSC and ESC removed revealed that the fibroblasts, ERC and BMSC formed three distinct groups, but the analysis did not separate ERC-E and ERC-B (Figure 4B). Unsupervised hierarchical clustering analysis also revealed the same cluster pattern according to cell type and there was no difference between ERC-B and ERC-E (Figure 4C).

Class comparison analysis was performed for the six cell types using BRB Array Tools (Ver 3.4.0), considering a p-value less than 0.001 as significant. Comparison of ERC-B and ERC-E showed 82 differentially expressed genes and a small fold change differences (data not shown). Due to small differences in gene expression between ERC culture under the two conditions, class comparisons and pathway analyses were subsequently conducted by comparing only ERC-B with the other groups. Almost three thousand genes (2974) were differentially expressed between ERC-B and fibroblasts, whereas 1030 genes were differentially expressed between BMSC and fibroblasts.

Between ERC-B and BMSC, 1923 genes were differentially expressed. Some of the most up-regulated genes in ERC-B when compared with BMSC included somatostatin receptor 1 (SSTR1), TNFSF4, coagulation factor 3 (F3), and MMP3 (Table 4) while the most down-regulated genes in ERC-B included prostaglandin I2 synthase (PTGIS), prostaglandin-endoperoxide synthase 2 (PTGS2), vascular cell adhesion molecule 1 (VCAM1), and integrin alpha 10 (ITGA10) (Table 4).

**Table 4:** Genes differentially expressed between ERC and BMSC

	Genes up-regulated in ERC*	**Fold-increase**		Genes down-regulated in ERC*	**Fold-decrease**
SSTR1	Somatostatin receptor 1	421.4	CYP1B1	Cytochrome P450, family 1, subfamily B	423.4
C3orf72	Chromosome 3 open eading frame 72	299.2	CYP1B1	Cytochrome P450, family 1, subfamily B, clone	258.4
F3	Coagulation factor III	182.5	IRX3	Iroquois homeobox 3	199.2
TNFSF4	Tumor necrosis factor superfamily, member 4	93.7	SLC14A1	Solute carrier family 14, member 1, transcript variant 3	172.8
FOXL2	Forkhead box L2	80.5	PITX2	Paired-like homeodomain 2	167.7
FAM105A	Family w/sequence similarity 105, member A	80.0	BAALC	Brain & acute leukemia, cytoplasmic, transcript var. 2	146.4
SYNPO2L	Synaptopodin 2-like, transcript variant 1	78.4	PTGIS	Prostaglandin I2 synthase	85.3
SLCO2A1	Solute carrier organic anion transporter family, member 2A1	64.6	IRX5	Iroquois homeobox 5	81.3
ANO4	Anoctamin 4	58.5	PRDM16	PR domain containing 16, transcript var. 2,	77.8
SPON2	Spondin 2, extracellular matrix protein, transcript var. 3	53.5	DLX5	Distal-less homeobox 5	72.4
CARD16	Caspase recruitment domain family, member 16, transcript var. 2	50.3	PTGS2	Prostaglandin-endoperoxide synthase 2 (prostaglandin G/H synthase and cyclooxygenase)	71.7
VAT1L	Vesicle amine transport protein 1 homolog-like	46.4	COLEC12	Collectin sub-family member 12	66.9
NBLA00301	Nbla00301 non-coding RNA	44.6	KRTAP1-1	Keratin associated protein 1-1	62.6
INMT	Indolethylamine N-methyltransferase, transcript var.2	42.8	TBX15	T-box 15	62.4
DIO2	Deiodinase iodothyronine type II, transcript variant 5	41.7	PDLIM3	PDZ and LIM domain 3, transcript variant 1	57.8
ALDH1A1	Aldehyde dehydrogenase 1 family, member A1	39.5	COMP	Cartilage oligomeric matrix protein	54.4
HTR2B	5-hydroxytryptamine (serotonin) receptor 2B	38.3	GAP43	Growth associated protein 43, transcript var. 2	53.0
CARD17	Caspase recruitment domain family member 17	35.9	VCAM1	Vascular cell adhesion molecule 1, transcript var.2	52.0
ARHGAP20	Rho GTPase activating protein 20	35.3	ITGA10	Integrin, alpha 10	50.2
ZBTB46	Zinc finger and BTB domain containing 46	34.3	LRRC15	Leucine rich repeat containing 15, transcript variant 2	48.9
SYNPO2L	Synaptopodin 2-like transcript variant 1	34.3	SLC14A1	Solute carrier family 14 (urea transporter), member 1, transcript variant 4	48.4
C13orf15	Chromosome 13 open reading frame 15	33.5	FLG	Filaggrin	48.2
HOXD11	Homeobox D11	32.5	DLX6	Distal-less homeobox 6	47.3
OLR1	Oxidized low density lipoprotein receptor 1, transcript var. 2	32.0	CMKLR1	Chemokine-like receptor 1, transcript variant 3	44.0
HOXD10	Homeobox D10	30.7	CH25H	Cholesterol 25-hydroxylase (CH25H), mRNA.	43.4
PSG4	Pregnancy specific beta-1-glycoprotein 4	30.5	PLAC9	Placenta-specific 9 (PLAC9), mRNA.	41.8
MMP3	Matrix metallopeptidase 3	29.2	PCOLCE2	Procollagen C-endopeptidase enhancer 2	41.5

P <0.0001 for all genes.

*The genes most up- and down-regulated according to fold-change are listed.

**Figure 5:**
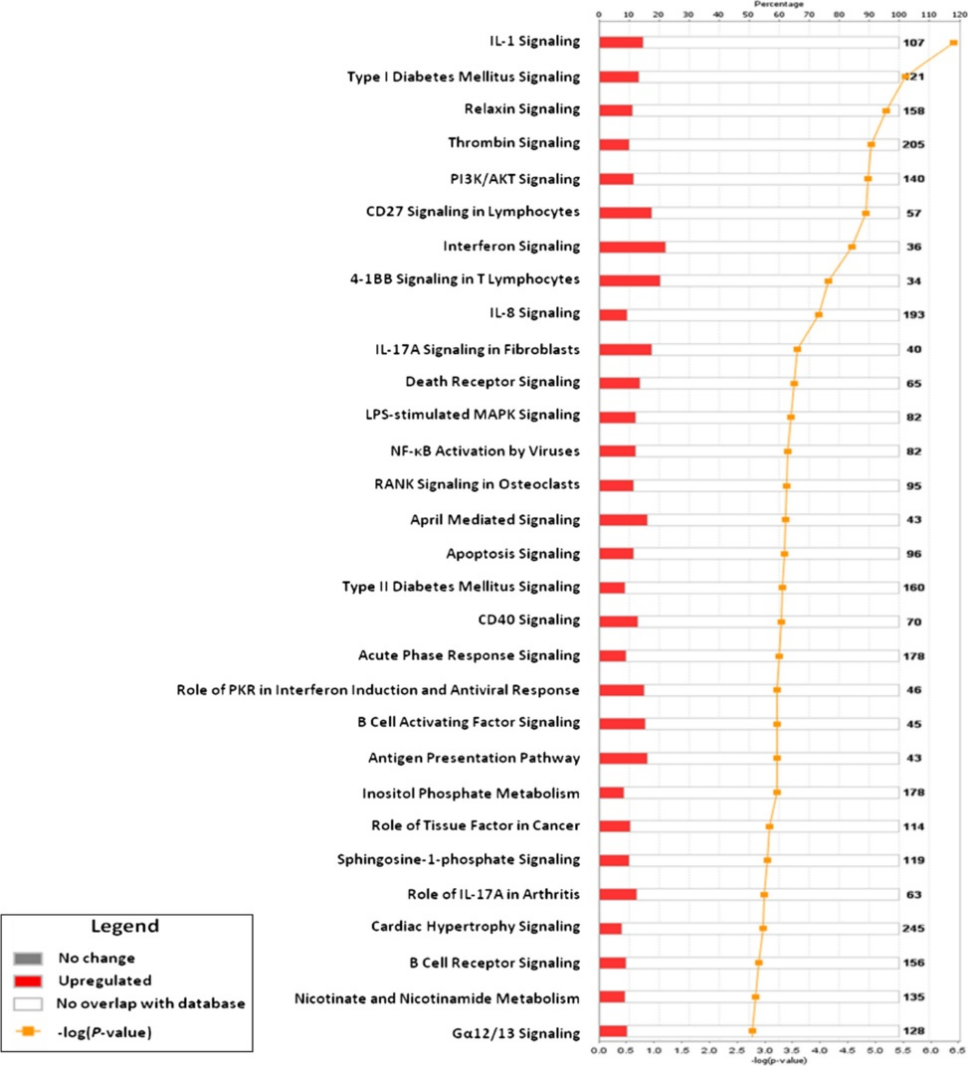
**Ingenuity pathway analysis of up-regulated genes in ERC-B samples compared with BMSC.** The 30 canonical pathways most significantly overrepresented with ERC-B up-regulated genes are shown (*P* < 0.05). The orange bar shows the ratio of the number of genes in each pathway divided by the total number of genes that make up that pathway. The yellow line indicated the significance of the ERC up-regulated genes in each pathway and is expressed as minus-log *P*-value.

**Figure 6:**
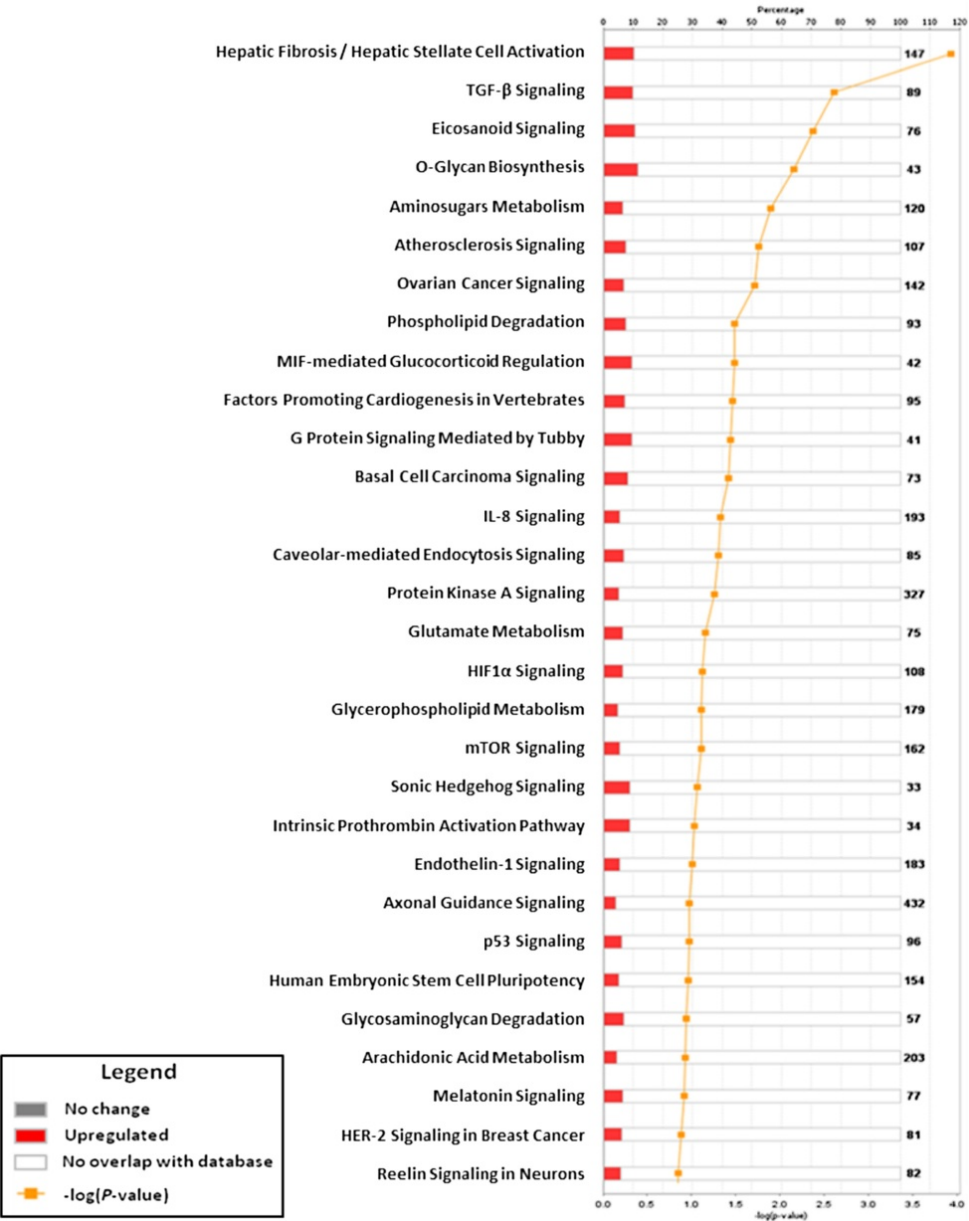
**Ingenuity pathway analysis of down-regulated genes in ERC-B samples when compared with BMSC.** The 30 canonical pathways most significantly overrepresented with ERC-B down-regulated genes are shown (*P* < 0.05). The orange bar shows the ratio of the number of genes in each pathway divided by the total number of genes that make up that pathway. The yellow line indicated the significance of the ERC-B down-regulated genes in each pathway and is expressed as minus-log P-value.

Ingenuity Pathway Analysis (IPA) was performed to annotate the differentially expressed genes between ERC and BMSC. This analysis revealed that the up-regulated genes in ERC-B were involved in many of the top canonical pathways, including pro-inflammatory pathways such as IL-1 and IL-8 signaling and immune response pathways such as interferon signaling, CD27 signaling in lymphocytes, B cell receptor signaling, and NF-κB activation by viruses, IL17a signaling, CD40 signaling and antigen presentation (Figure 5). Pathways over represented among genes down-regulated in ERC-B were Hepatic fibrosis/hepatic stellate cell activation, TGF-B signaling, atherosclerosis signaling, mTor signaling, human embryonic stem cell pluripotency and hedgehog signaling and the cancer signaling pathways, ovarian cancer signaling, basal cell carcinoma and HER-2 signaling in breast cancer (Figure 6).

### Biologically relevant genes preferentially expressed in ERC vs BMSC

Analysis of genes preferentially expressed in ERC vs BMSC based on biological significance was performed manually. Several genes of interest were identified. FoxL2 transcript, a transcription factor essential for ovary development [20] was expressed 80.5-fold higher in ERC compared to BMSC (Table 5). Mindin (SPON2) transcript, an innate immunity receptor involved in bacterial recognition [21] was expressed 53.5-fold higher as compared to BMSC. The stem cell potency marker, aldehyde dehydrogenase [22] was expressed at transcript level 39.5-fold higher. Immune modulatory proteins pregnancy associated glycoprotein 1 [23] and GM-CSF [24] were expressed 30.5- and 5.0-fold higher. Angiogenesis associated proteins Angiopoietin-1 [25] and PDGF [26] were expressed 13.8- and 26.9-fold higher. The matrix metalloprotease 3 [27] transcript was 29.2-fold higher expressed as compared to BMSC. Interestingly, a comparison of MSC to ERC by Meng et al. revealed that ERC possessing substantially higher protein production of Angiopoietin, PDGF, GM-CSF and MMP3 as compared to MSC [14].

**Table 5:** Biologically-Relevant mRNA Expression Compared Between ERC and BMSC

**Fold Higher Expression in ERC**	**Gene**	**Property**	**Reference**
80.5-fold	FoxL2	Female-specific gonadal differentiation transcription factor	[20]
53.5-fold	Mindin (SPON2)	Bacterial recognition receptor	[21]
39.5-fold	Aldehyde dehydrogenase	Associated with stem cell potency in hematopoietic stem cells, angiogenic stem cells and cancer stem cells	[22]
30.5-fold	Pregnancy associated glycoprotein 1	Anti-inflammatory product that induces IL-10, protects fetus from maternal immune system	[23]
29.2- fold	Matrix Metalloprotease 3	Tissue remodeling, angiogenesis	[27]
26.9-fold	PDGF	Angiogenesis, endothelial survival, vessel maturity	[26]
13.8-fold	Angiopoietin	Stimulates angiogenesis	[25]
5.1-fold	GM-CSF	hematopoiesis/inhibits autoimmuity	[24]

### Quantitative RT-PCR results

**Figure 7:**
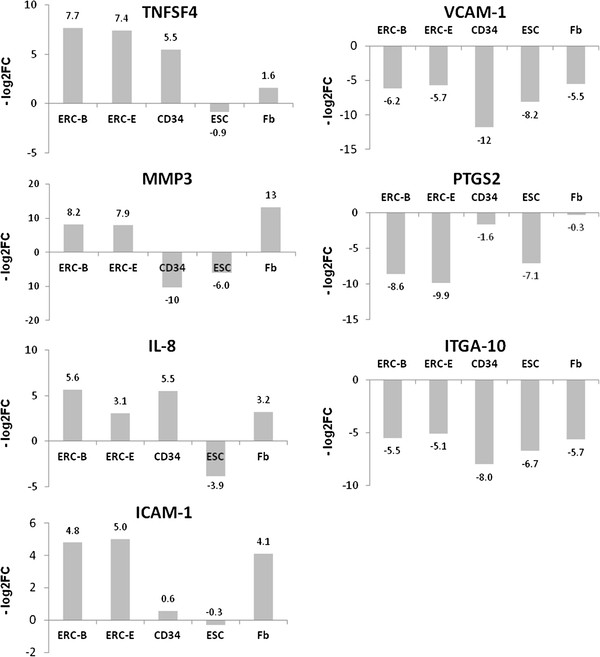
**Measurement of the expression of selected genes among 6 ERC-B, 6 ERC-E, 3 HSC (CD34), 3 ESC, and 3 fibroblast samples using q RT-PCR.** The expression levels of TNFSF4, MMP3, IL-8 and ICAM-1 were up-regulated in ERC compared to BMSC and the expression of VCAM-1, PTGS2 and ITGA-10 were down-regulated in ERC. The fold change values shown were normalized against BMSC and calculated as the –log_2_fold-change (−log_2_FC).

Quantitative RT-PCR analysis was performed to confirm the expression of differentially expressed genes between ERC-B and BMSC. The raw data was calculated based on an 18sRNA endogenous control, and log2-transformed fold change against the BMSC was calculated based on the ΔΔCt values. Fold changes between ERC-B and ERC-E for most genes were less than 2-fold with the exception of IL-8 and PTGS2, which had 2.5-fold and 1.3-fold differences, respectively confirming that there was little difference in ERC grown in the different media (Figure 7). In addition, the expression of TNFSF4, MMP3, IL-8, and ICAM-1 was several fold greater in ERC with respect to BMSC, while the expression VCAM-1, PTGS2, ITGA-10 was several fold lower in ERC than in BMSC. These data were in good accordance with our microarray finding.

### miRNA microarray analysis

**Figure 8:**
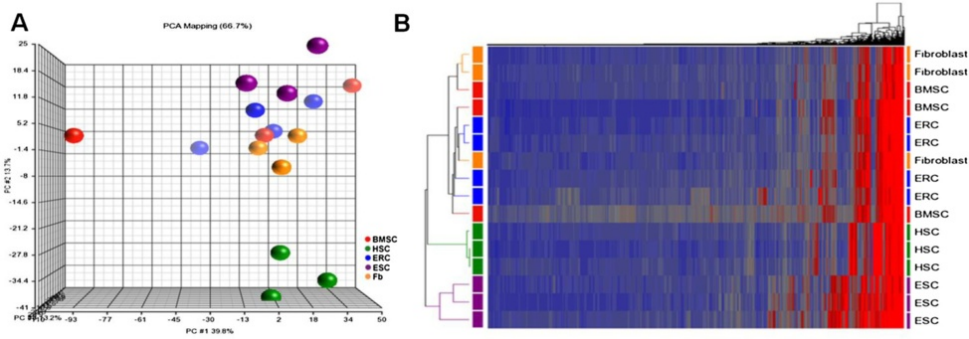
**Principal component and hierarchical clustering analysis of miRNA differentially expressed among BMSC, ERC-B, HSC, ESC, and fibroblast (fb) samples.A**: Principal component analysis of all five cell types based on differential expressed miRNA. **B**: Hierarchical clustering analysis of all five cell types based on differentially expressed miRNA.

A miRNA profiling was performed on ERC-B, BMSC, fibroblasts, ESC, and HSC. PCA analysis showed three distinct clusters. The BMSC, ERC, and fibroblasts were again closely grouped together, and they were also clustered close to the ESC samples (Figure 8-A). HSC were in a third distinct group of their own. The hierarchical clustering analysis of the samples also showed that BMSC, ERC, and fibroblasts formed a mixed cluster, instead HSC and ESC samples formed two separate clusters (Figure 8-B).

## Discussion

The increased interest in MSC for therapeutic applications in recent years has led to many new discoveries of regenerative cells from different origins. In addition to ERC and BMSC, other MSC have been used clinically or considered for clinical use and the group includes those derived from adipose tissue and umbilical cord blood. There have been some comparisons of the different types of stromal cells but this is the first comparison between ERC and BMSC both of which are being tested in clinical trials.

BMSC have been compared with other types of stromal cells including adipose tissue, lung tissue and umbilical cord blood derived MSC [28-32]. These studies have found that stromal cells produced from these various cell and tissue types have a similar phenotype [28-32]. All express CD44, CD73, CD90 and CD105, but do not express hematopoietic cell markers. They all demonstrate some degree of osteoblastic, chondrogenic and adipogenic differentiation ability, but there were some differences in differentiation ability among them [30,31], particularly when expression of differentiation markers were compared [29,32,33]. In addition the proliferation of stromal cells derived from diverse sources sometimes differ [28,31]. Stromal cells derived from different tissues can modulate immune function, but the degree of immune modulation can vary. For example, BMSC and adipose tissue derived-MSC differ in their ability to modulated the production of immunoglobulin by mitogen-stimulated B cells [30] and the proliferation of allogeneic T cells [34]. Some studies have also found differences in the expression of genes encoding cytokines and growth factors among rat adipose tissue MSC, rat cartilage MSC and rat BMSC [33]. None of these studies have compared global gene or miRNA expression among different types of stromal cells. The results of our study were similar to previous reports in that BMSC and ERC had similar morphology and proliferation, but we found some differences in gene expression in addition to the differences in cytokine and growth factor production.

We noted morphological differences between ERC and BMSC, but both cells resemble fibroblasts and morphology differences have also been noted between other types of MSC [34]. The PDTs of ERC and BMSC were similar, with BMSC having a slightly shorter PDT. Our PDT findings for ERC differed from those found from earlier literature, which was reported to be around 19 hours. This difference may be due to slight variations to the culture media formula and culture conditions. Immunofluorescence staining showed that both ERC and BMSC were positive for the pluripotency marker Oct-4, which is consistent with findings from earlier studies [14]. Comparison of miRNA expression signatures also found that ERC were very similar to BMSC.

Gene expression analysis revealed considerable similarities between ERC and BMSC, but there were also distinct differences between these two types of cells. Many immunity and inflammation related pathways were overrepresented among genes differentially expressed between ERC and BMSC suggesting that they may have different immune modulatory and anti-inflammatory properties. This is supported by qRT-PCR analysis which showed that the expression of TNFSF4, MMP3, IL-8 and ICAM-1 were greater in ERC, and that of VCAM-1, PTGS2 and ITGA10 were greater in BMSC. Comparison of the levels of cytokines and growth factors in ERC and BMSC supernatants also found difference among ERC and BMSC. The levels of IL-8 and ICAM-1 were greater in ERC supernatants and the levels of HGF, VEGF, TGFB2 and IL-6 were greater in BMSC supernatants. We found slight differences in the inhibition of MLRs between ERC and BMSC; the ERC being slightly more inhibitory. Interesting, adipose derived MSC have been found to be more immunomodulatory than BMSC [30,34]. In addition, the expression of two factors important in angiogenesis differed among these two types of stromal cells; MMP-3 was greater in ERC at the transcript level and VEGF was greater in BMSC supernatants at the protein level. This suggests the ability of ERC and BMSC to support angiogenesis may also differ. Specific analysis of biologically relevant genes that were overexpressed in ERC revealed substantially higher expression of genes associated with angiogenesis, including PDGF, and Angiopoietin-1. This is of interest since a previous publication by Meng et al., reported higher protein expression of MMP-3, angiopoietin, and PDGF [14].

Global gene and miRNA expression analysis found that fibroblasts were similar to ERC and BMSC. This is consistent with the fact that, like ERC and BMSC,, fibroblasts express CD73 and CD105 and lack hematopoietic markers CD14, CD34 and CD45 and they demonstrate osteogenic, chondrogenic and adipogenic differentiation [35,36]. They also have immunosuppressive properties. They can inhibit mitogen and allogeneic stimulated T cell proliferation and interferon-γ production [35,37].

The results of this study show that although ERC and BMSC are currently being tested in clinical trials for similar indications, they may not have identical clinical effects. While our studies found a number of differences in gene expression, protein production and in vitro function which suggest that these two cell types may differ in their immunomodulatory and anti-inflammatory effects, we cannot tell from these studies how they will perform in vivo or which cell type will have greater immunomodulatory or anti-inflammatory effects. We suspect that multiple properties of stromal cells are responsible for their in vivo effectiveness and ERC may be more effective for some of the clinical applications and BMSC for others. Studies in animal models or clinical trials will be required to more fully characterize the differences between ERC and BMSC.

## Conclusions

While the miRNA and gene expression signatures of ERC and BMSC are very similar, we found some differences in a number of immune and inflammatory pathways at the transcriptome and protein levels. The ability of ERC and BMSC to inhibit MLR also differed slightly. This suggests that the in vivo effects of these two types of MSC may also differ.

## Competing interests

TEI and VB are employees and shareholders of Medistem (MEDS: Pinksheets) which produced the ERC.

## Authors’ contributions

HW helped design the study, performed experiments, analyzed data and wrote the manuscript. PJ assisted with the study design, preformed experiments, analyzed data analysis and helped write the manuscript. SC performed experiments and analyzed the data. MS helped design the study, analyzed the data and wrote the manuscript. JR provided the BMSC and preformed experiments and analyzed the data. DFS, TEI and VB conceived and helped design the studies. TEI and VB provided the ERC. DFS and TEI reviewed that data and helped write the manuscript. All of the authors read and approved the final manuscript.
